# Application of Community-Based Participatory Research to Support Chinese American Dementia Caregivers

**DOI:** 10.1093/geroni/igaf122.4367

**Published:** 2025-12-31

**Authors:** Jinyu Liu, Ada Yuk-Sim Mui, Ethan Siu Leung Cheung, Yifan Lou, Qin Sun

**Affiliations:** Baylor University, Waco, Texas, United States; Columbia University, New York, New York, United States; The University of Hong Kong, Hong Kong; Virginia Commonwealth University, Richmond, Virginia, United States; Baylor University, Waco, Texas, United States

## Abstract

**Objectives:**

This study aims to apply community-based participatory research (CBPR) to develop and evaluate a culturally sensitive intervention to reduce the stress of Chinese American dementia caregivers, an understudied population in implementation science.

**Methods:**

Guided by CBPR principles, we used multiple strategies for partnership formation, community assessment, intervention development, feasibility evaluation, and intervention improvement.

**Results:**

We formed a CBPR team including administrators, social workers, and caregivers from the community and investigators and research assistants. Using mixed methods, we assessed the needs and strengths of Chinese ADRD caregivers through 30 interviews and a survey (N = 165). Based on assessment results, the CBPR team developed a peer mentoring program engaging former caregivers to support current ones. A pilot test showed high feasibility and potential to reduce loneliness and depression while enhancing mentors’ sense of purpose. The team has refined the intervention for further evaluation.

**Discussions:**

CBPR was useful to facilitate trust-building, cultural tailoring, and shared decision-making for intervention development grounded in caregivers’ experiences and strengths. This study contributes to the growing evidence supporting CBPR as a powerful framework for advancing racial minority’s engagement in health studies and intervention development. Through deep community engagement, the project addressed both the unmet needs and unique strengths of an underserved population. Our findings emphasize that CBPR not only enhances research participation and intervention relevance, but also fosters mutual learning, community empowerment, and sustainable support systems. research should expand on this work by applying and tailoring CBPR to other social and cultural contexts to support diverse caregiving communities.

